# Nanoporous Alumina Support Covered by Imidazole Moiety–Based Ionic Liquids: Optical Characterization and Application

**DOI:** 10.3390/nano12234131

**Published:** 2022-11-23

**Authors:** Manuel Algarra, Mª Cruz López Escalante, Mª Valle Martínez de Yuso, Juan Soto, Ana L. Cuevas, Juana Benavente

**Affiliations:** 1 INAMAT^2^-Institute for Advanced Materials and Mathematics, Departamento de Ciencias, Universidad Pública de Navarra, Campus de Arrosadía, 31006 Pamplona, Spain; 2 Departamento de Ingeniería Química, Facultad de Ciencias, Universidad de Málaga, 29071 Málaga, Spain; 3 X-ray Photoelectron Spectroscopy Lab., Central Service to Support Research Building (SCAI), University of Málaga, 29071 Málaga, Spain; 4 Departamento de Química-Física, Facultad de Ciencias, Universidad de Málaga, 29071 Málaga, Spain; 5 Unidad de Nanotecnología, Centro de Supercomputación y Bioinnovación, Servicios Centrales de Investigación, Universidad de Málaga, 29071 Málaga, Spain; 6 Departamento de Física Aplicada I, Facultad de Ciencias, Universidad de Málaga, 29071 Málaga, Spain

**Keywords:** nanoporous alumina, ionic liquids, optical properties, solar cell

## Abstract

This work analyzes chemical surface and optical characteristics of a commercial nanoporous alumina structure (NPAS) as a result of surface coverage by different imidazolium-based ionic liquids (1-butyl-3-metylimidazolium hexafluorophosphate, 3-methyl-1-octylimidazolium hexafluorophosphate, or 1-ethyl-3-methylimidazolium tetrafluoroborate). Optical characteristics of the IL/NPAS samples were determined by photoluminescence (at different excitation wavelengths (from 300 nm to 400 nm), ellipsometry spectroscopy, and light transmittance/reflectance measurements for a range of wavelengths that provide information on modifications related to both visible and near-infrared regions. Chemical surface characterization of the three IL/NPAS samples was performed by X-ray photoelectron spectroscopy (XPS), which indicates almost total support coverage by the ILs. The IL/NPAS analyzed samples exhibit different photoluminescence behavior, high transparency (<85%), and a reflection maximum at wavelength ~380 nm, with slight differences depending on the IL, while the refractive index values are rather similar to those shown by the ILs. Moreover, the illuminated I–V curves (under standard conditions) of the IL/NPAS samples were also measured for determining the efficiency energy conversion to estimate their possible application as solar cells. On the other hand, a computational quantum mechanical modeling method (DFT) was used to establish the most stable bond between the ILs and the NPAS support.

## 1. Introduction

Room temperature ionic liquids (RTILs or ILs) are compounds of great interest nowadays in a broad spectrum of research areas (from electrochemistry and energy to catalysis and green chemistry) due to their significant properties [1,2]. As it is well known, ILs are typically comprised of an organic cation paired with an inorganic/organic anion, and they exhibit very low vapor pressure and remain in the liquid state at temperatures below 100 °C. ILs are non-flammable compounds, and they possess very interesting properties such as low volatility, good electrical conductivity and wide electrochemical windows, high chemical and thermal stability, and good ability to dissolve a wide range of compounds; consequently, they are considered as an alternative to volatile organic solvents (green solvents) [3,4]. Moreover, the physicochemical properties of ILs can be modified by tuning the structure of the cation and/or the anion, which allows the selection of the cation/anion combination more adequate for a given application, increasing their use as heat transfer fluids and energetic materials or in extraction and separation processes, catalytic and biochemical reactions, electrochemical devices (batteries, capacitors, fuel cells or solar cells), or gas sensors (based on the optical properties of the ILs) [5,6,7,8,9,10,11,12,13,14,15,16].

Among the variety of ILs, those based on imidazolium (1-methyl-3-butylimidazolium tetrafluoroborate, 1-methyl-3-butylimidazolium bromide, 1-ethyl-3-methylimidazolium tetrafluoroborate, 1-dodecyl-3-methylimidazolium chloride, 1-methyl-3-butylimidazolium hexafluorophosphate, etc.) are commonly used in most of the previously indicated applications. In addition, among other optical characteristics, interesting fluorescence behaviors for 1-ethyl-3-methylimidazolium tetrafluoroborate and 1-butyl-3-metylimidazolium hexafluorophosphate (attributed to imidazolium moiety) were already reported [17,18], which opens the field of application of this type of ILs to those areas where fluorescence probes are involved. On the other hand, it should be pointed out that their liquid state can make difficult to handle ILs in some applications (energy storage, catalysis, or lubrication), their immobilization being necessary in solid structures such as polymeric matrices (ionic gels) or films, where IL/biopolymer hybrid materials are emerging as biomaterials in biomedical applications [19] and also in carbon nanomaterials (nanotubes and nanofibers), graphene, electrospun nanofibers, silicone matrix, or other nanoporous structures (ceramic supports) for handling purposes and also for improving system performance [20,21,22]. In particular, incorporation of imidazolium-based ILs in the well-defined pores of metal-organic frameworks (MOFs) has been recently studied [23], and, as a result of nanopore confinement, supercapacitors with high capacitances and energy densities were obtained, while disposable IL-sensors were obtained using thin alumina layer coated plates, silica, cotton, or paper supports [24]. Furthermore, the fluorescence increases for IL-filled micro reservoirs as well as the stability and durability of imidazolium-based ILs immobilized on porous and non-porous inorganic materials already reported [25,26] are other points of interest. In this context, although immobilization/confinement of ILs is in many cases a strategy to overcome manageability problems, interactions between ILs and the support could also exist. In fact, possible changes in the structure and physicochemical properties of ILs when they are nanoconfined into negatively charged surfaces (pores or films) have already been reported [27], indicating the effect of solid surface chemistry and charge, temperature, and ion type on the layered structure of the IL adjacent to the solid surface.

Nanoporous alumina structures (NPASs) obtained by electrochemical anodization of aluminum foils using the two-step anodization method [28] are a kind of nanoporous supports of great interest nowadays due to their good chemical and thermal stability, large specific surface area, adequate hardness, and structural regularity since they usually present parallel cylindrical straight pores (without tortuosity and very narrow pore radius distribution) [29,30]. NPASs, which exhibit positive effective fixed charge in contact with aqueous solutions [31], have an average pore radius ranging between 10 nm and 200 nm, with interpore distance between 65 nm and 400 nm, depending on the applied voltage, electrolyte solution, and temperature used during the first anodization step (in case of symmetrical samples), while porosity, another important geometrical parameter, commonly ranges between 10% and 30% [32,33]; however, asymmetric NPASs can also be obtained by sequential modification of electrochemical conditions [34]. In fact, the possibility of obtaining commercial NPASs as well as the easy surface characteristic modification by the atomic layer deposition technique [35,36,37] facilitates their use as support platforms for diverse applications.

In this work, the optical characterization of three samples obtained by deposition of imidazolium-based ILs layer on a commercial NPAS is performed (IL/NPAS samples). The selected ILs were 1-butyl-3-metylimidazolium hexafluorophosphate, 3-methyl-1-octylimidazolium hexafluorophosphate, and 1-ethyl-3-methylimidazolium tetrafluoroborate since they allow us to consider the effect of aliphatic chain length or anion on sample optical parameters, which could be of interest for optical sensor applications [15]. Chemical surface characterization of the ILs/NPAS was performed by X-ray photoelectron spectroscopy (XPS) to determine NPAS coverage and/or to detect the presence of impurities, while a computational quantum mechanical modeling method was used to establish the most stable bond between the ILs and the NPAS. The optical characteristics of the IL/NPAS samples were determined by photoluminescence (for excitation wavelength between 300 nm and 400 nm), ellipsometry spectroscopy, and light transmittance/reflectance (wave ranging between 200 nm and 1700 nm, providing information on visible and near-infrared regions) measurements. Moreover, I–V curves were also determined for possible solar cell application of the analyzed IL/NPAS systems. These results show the photoluminescence character of the three IL/NPAS samples, as well as their high transparency (>85%) and a reflection maximum at wavelength ~380 nm, with slight differences depending on the IL, while the refractive index values determined for a wide range of wavelengths do not differ significantly from those corresponding to the ILs analyzed. Further, considering the application of the IL/NPAS samples as solar cells, an increase between 10% and 25% in efficiency, depending on the IL, was determined.

## 2. Materials and Methods

### 2.1. Materials

**Figure 1:**
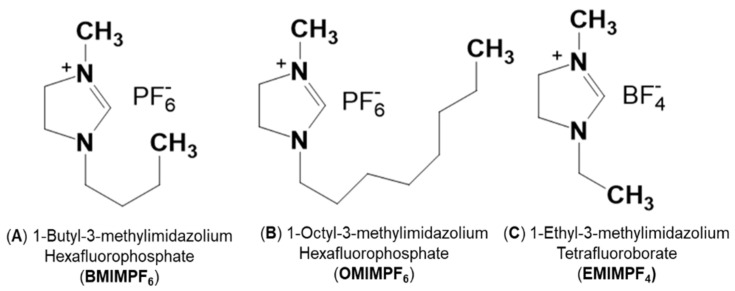
Molecular structure of the protic ionic liquids used in this study (ChemWindow Chemical Structure Drawing Software from Wiley).

Protic ionic liquids based in imidazole moieties 1-Ethyl-3-methylimidazolium tetrafluoroborate (C_6_H_11_N_2_:BF_4_ (EMIMBF_4_); ≥98% HPLC grade); 1-Butyl-3-metylimidazolium hexafluorophosphate (C_8_H_15_N_2_:PF6 (BMIMPF_6_); ≥97% HPLC grade); and 1-octyl-3-methyl-imidazolium hexafluorophosphate (C_12_H_23_N_2_:PF_6_ (OMIMPF_6_); ≥97% HPLC grade) were purchased from Sigma-Aldrich (Barcelona, Spain) and used without further purification. Figure 1 shows the molecular structures of the studied ILs, while Table 1 indicates some basic physicochemical characteristics. Although the three selected ILs have an aromatic ring, they exhibit different molar volume and viscosity as well aliphatic chain length and even anions, which can influence the corresponding IL/support interactions.

### 2.2. Preparation of the NPAS Modified by ILs Samples

A commercial asymmetric nanoporous alumina structure (NPAS) from Whatman (Anodisc^TM^, Sigma-Aldrich, Barcelona, Spain) was used as support. Characteristic NPAS geometrical parameters are 60 μm thickness, 10 nm nominal pore radius for one surface (surface A) and 100 nm nominal pore radius for the other surface (surface B), and porosity around 23% (surface A) or 35% (surface B) [39]. A certain amount of each IL was plugged on the denser surface (A) of the NPAS for its coverage and these samples are hereafter named as BMIMPF_6_/NPAS, OMIMPF_6_/NPAS, and EMIMBF_4_/NPAS. The commercial character of both components of the studied samples reduces preparation time and cost.

### 2.3. Chemical Surface Analysis

Chemical characterization of sample surfaces was performed by analyzing X-ray photoelectron spectroscopy (XPS) spectra, which were obtained with a Physical Electronics Spectrometer (PHI 5700, Lake Drive East, Chanhassen, MN, USA) with X-ray MgK_α_ radiation (15 kV, 300 W and 1253.6 eV) as the excitation source. A concentric hemispherical analyzer, operating in the constant pass energy mode at 29.35 eV, was used for recording the high-resolution spectra at a take-off angle of 45°. The diameter of the analyzed area was 720 mm and each spectral region was scanned several times to have a good signal (low noise contribution). The residual pressure in the analysis chamber was maintained below 5 × 10^−7^ Pa during data acquisition, and binding energies (accurate ± 0.1 eV) were determined with respect to the position of the adventitious C 1*s* peak at 285.0 eV. Shirley-type background and Gauss–Lorentz curves were used to determine the binding energies following the methodology described in detail elsewhere [40]. On the other hand, although XPS measurements were performed with a conventional (ultra-high-vacuum) spectrometer, its suitability for the characterization of ionic liquids has already been demonstrated [41,42].

### 2.4. Quantum Chemical Calculations

The interactions of the imidazole derivatives with the nanoporous alumina structure were computationally studied by means of density functional theory (DFT). The hybrid exchange-correlation functional CAM-B3LYP [43] and the def2-SVPP basis sets [44,45] were applied to all the atoms which conform the systems under study, while the electronic structure calculations were carried out with the program GAUSSIAN16 [46]. The potential energy surfaces which lead to the formation of the complexes formed between imidazole derivatives and NPASs were studied by means of the linear interpolation method [47,48,49]. Molecular geometries were analyzed with the help of the MacMolPlt [50] and MOLDEN [51] graphical programs.

### 2.5. Optical Characterizations

An Edinburgh Instruments FLS920, equipped with a Xe lamp (450 W) as excitation source and monochromatic LEDs (PicoQuant PLS, Berlin, Germany), controlled by a PDL880-B system, was used for steady state fluorescence measurements.

Transmittance/reflection measurements were performed with a Varian Cary 5000 spectrophotometer (Agilent Technologies, Santa Clara, CA, USA) provided with an integrating sphere of Spectralon for wavelength ranging between 250 and 2000 nm.

Spectroscopic ellipsometry (SE) measurements were carried out with unsupported samples using a spectroscopic ellipsometer (Sopra-Semilab GES-5E, Budapest, Hungary) at an incident angle Φ_o_ = 70° for wavelengths ranging between 200 nm and 1700 nm. WinElli software v.2.2 from Sopra-Semilab was used for data analysis and fittings. Spectroscopic ellipsometry is a non-destructive and contactless technique used to characterize single layers or a small number of well-defined layers that are optically homogeneous and isotropic. SE measures the change of light polarization upon reflection or transmission by means the Ψ and Δ angles [52]. Supplementary Information shows a scheme of incident, transmitted, and reflected light for a two-layer system (Figure S1a) and indication of the different parameters involved in SE measurements and the relation among them (Figure S1b).

### 2.6. I–V Curve Measurements

I–V curves were measured in an ABA-class LED solar simulator (LSH-7320) from Oriel S.A., under standard conditions (1000 W/m^2^ irradiance at 25 °C and incident spectrum AM1.5G), according to IEC 60904-9 [53]. A commercial monocrystalline silicon solar cell of 156 mm side was used, although the radiation of the solar simulator only reaches a circular area of 2 cm in diameter. I–V curves for the three IL/NPAS samples, in addition to the NPAS support, were measured. To reduce measurement uncertainty, the I–V curve for each system was measured four times maintaining the solar cell area, and average values of the estimated parameters are provided.

## 3. Results and Discussion

### 3.1. Chemical Surface Analysis

**Figure 2:**
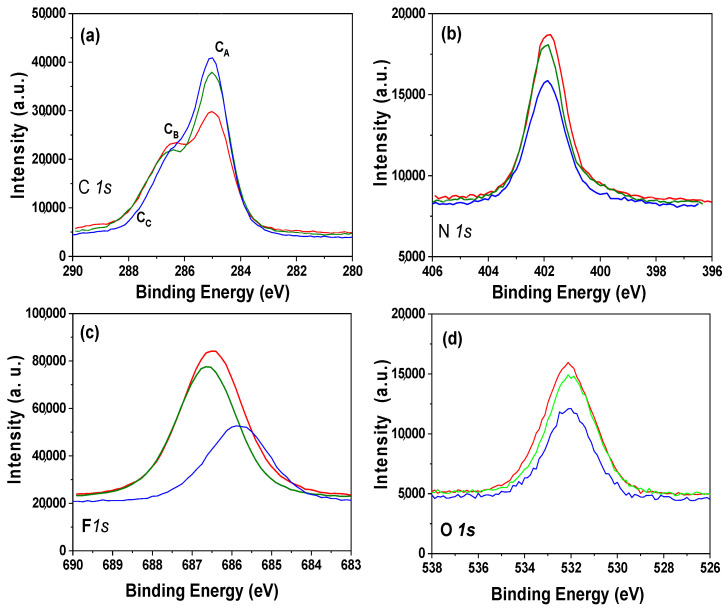
Core level signals for BMIMPF_6_/NPAS (red line), OMIMPF_6_/NPAS (green line), and EMIMBF_4_/NPAS (blue line) samples. (**a**) C *1s*; (**b**) N *1s*; (**c**) F *1s*; (**d**) O *1s*. Dashed black line corresponds to NPAS support.

Chemical surface characterization of the alumina nanoporous structure coated with the different ILs was performed analyzing the XPS spectra. The survey spectra obtained for the IL/NPAS samples, which allows us to identify the elements present on the samples surface, is shown as Supplementary Information (Figure S2), and peaks associated with the characteristic elements of the studied ILs (C, N, F, and P for BMIMPF_6_ and OMIMPF_6_ or C, N, F, and B for EMIMBF_4_) can be observed, but other elements related to sample support or contamination (Al and O) were also detected. The atomic concentration percentage (A.C. %) of the elements found on the surfaces of the analyzed IL/NPAS samples were determined by the areas of the corresponding core level signals (from narrower detailed scans of the selected peaks) as those shown in Figure 2. In particular, Figure 2a shows a comparison of the carbon C *1s* core level for the three IL/NPAS samples, where a clear peak (C_A_) at a binding energy of 285.0 eV (assigned to the alkyl chain of the ILs and adventitious carbon [54]) can be observed, plus a shoulder at 286.5 eV (C_B_) assigned to imidazolium C = N bonds [54], but another small shoulder at around 287.5 eV associated with the C = O bond from surface oxidation or contamination/impurities is also detected. The N *1s* signal (Figure 2b) presents a rather symmetric peak at a B.E. of 401.9 eV for the three IL/NPAS samples, assigned to the C = N bonds from the imidazolium [55]. In the case of the F *1s* core level (Figure 2c), three symmetric peaks can also be observed, with a maximum at the B.E. of 686.5–686.8 eV for the BMIMPF_6_/NPAS and the OMIMPF_6_/NPAS samples, which corresponds to the PF_6_^−^ bond, but at 685.9 eV in the case of the EMIMBF_4_/NPAS sample (BF_4_^−^ bond) [56]. Figure 2d shows the O 1s core level signal for the three IL/NPAS samples as well as for the NPAS support (NPAS intensity was reduced 60% for scaling reason), where a peak at the B.E. of 532.0 eV, associated with the support, can be observed. B 1s spectrum for the EMIM.BF_4_/NPAS and P 2*p* spectrum for BMIMPF_6_/NPAS and OMIMPF_6_/NPAS samples are provided as Supplementary Information (Figure S3a,b), and practically no difference can be observed for these latter samples.

Table 2 shows the atomic concentration percentage of the elements found on the surface of the analyzed IL/NPAS samples and, as it can be observed, the surfaces of all of them also exhibit alumina and oxygen (even phosphorous in the case of sample EMIMBF_4_/NPAS), elements characteristic of the alumina support, as well as carbon and phosphorous (see Table S1 in Supplementary Information) associated with the manufacturing procedure [57]. Table 2 shows a comparison of experimental (E) and theoretical (T) ratio values (in italics) of some elements, and these results indicate an excess of carbon on the surface of all the samples (according to the C/N and C/F ratios), which could be associated with environmental contamination (or even with the NPAS support), being more significant in the case of the EMIMN_2_BF_4_/NPAS; in fact, this sample exhibits more impurities and, consequently, higher difference among experimental and theoretical values. On the other hand, the reduction in the experimental F/P and F/B ratios could be related to a preferential orientation of the ILs in the vicinity of the alumina support.

### 3.2. Interactions of Imidazole-Moieties with NAPS

**Figure 3:**
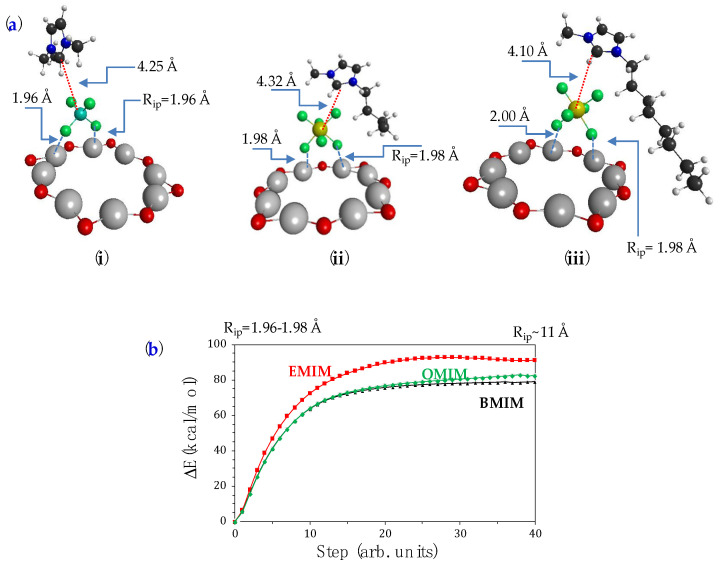
(**a**) CAM-B3LYP/def2-SVPP minimum energy geometries of IL/NAPS systems with (i) EMIMBF4; (ii) BMIMPF6; (iii) OMIMPF6. (**b**) Potential energy profiles of the imidazole moiety/NPAS systems formation-dissociation processes.

The structural models chosen for studying the interaction of the imidazole derivatives with NAPSs consist of a star-shaped (Al-O)_8_ geometry with two positive charges (Figure 3a) in analogy with that proposed by Zamani [58] and the corresponding imidazole. Thus, in accordance with the structural models selected in this work, whose minimum energy geometries are represented in Figure 3a, we found that the interactions of the imidazole derivatives with the alumina ring correspond to the formation of noncovalent bonds (van der Waals bonding) between imidazole-anion-NAPS whose origins are purely coulombic [59]. It must be noted that the inorganic anion always attaches to the border of the cavity. Table 3 collects the electronic adsorption energies (Δ_a_E) computed from the quantum chemical calculations. The less stable sample (or system) corresponds to BMIMPF_6_/NAPS. In addition, we studied the topology of the potential energy surfaces (PES) that led to the formation of each molecular complex (Figure 3b), and they were obtained with a linear interpolation method [60,61,62] which gives a reliable representation of PES within the internal coordinate space that has been selected in each complex. Such interpolations indicate that the potential surfaces for the formation of the complexes are barrierless; that is, the binding of the ionic pair to the alumina is exothermic.

### 3.3. Optical Characterization

**Figure 4:**
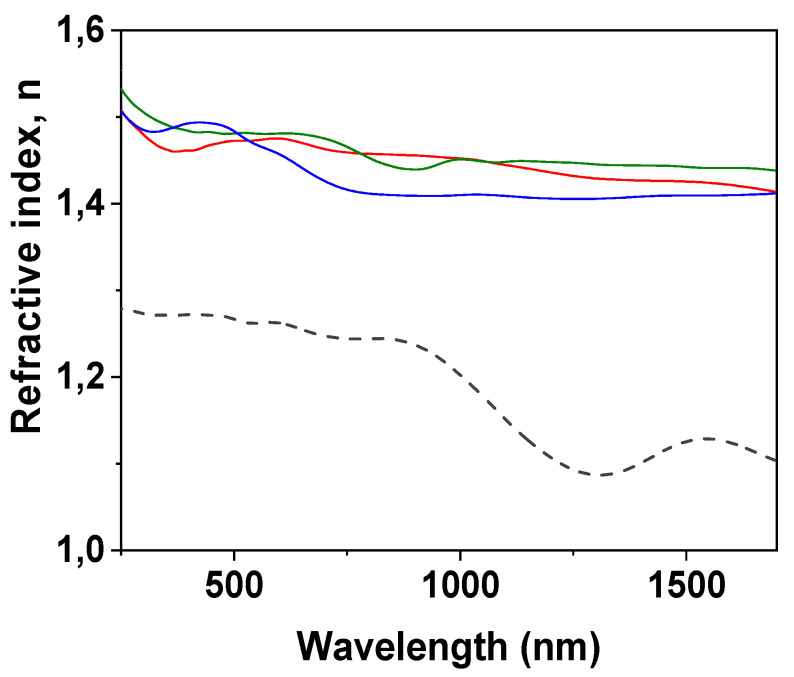
Wavelength dependence of refraction index for BMIMPF_6_/NPAS (red solid line), OMIMPF_6_/NPAS (green solid line), and EMIMBF_4_/NPAS (blue solid line) samples and the NPAS support (black dashed line).

Information on optical parameters of the studied samples was obtained from spectroscopic ellipsometry (SE) and light transmission/reflection measurements, which are two non-destructive techniques used commonly for the characterization of layered and/or modified thin films [63,64]. Figure 4 shows the wavelength dependence of the refractive index (n) determined for the IL/NPAS samples, which were obtained from spectroscopic ellipsometry (SE) experimental parameters (angles Ψ and Δ) taking into account the ellipsometer software and the variation of tanΨ and cosΔ with wavelength for the three IL/NPAS is presented as Supplementary Information (Figure S4); for comparison, n values for the NPAS [39] are also indicated in Figure 4. Average values of the refractive index determined for the three IL/NPAS for both optical regions (visible and nir) are indicated in Table 4. As it can be observed, the values of the refractive index obtained for the IL/NPAS samples do not differ significantly from the tabulated values previously indicated in Table 1 (4% increase considering the visible region) and following the same sequential order; however, these values are significantly higher (~28%) than those determined for the NPAS support (<n_visible_> = 1.277 ± 0.03 and <n_nir_> = 0.82 ± 0.23 [39]), which can be taken as a confirmation of adequate surface coverage by the corresponding IL. In this context, it should be indicated that the tabulated values of the ILs refractive index are usually determined at a given wavelength (or discrete number of wavelengths) for the visible region, covering these results a wider range of wavelengths. On the other hand, the significant effect of NPASs geometrical parameters on refractive index should be pointed out: porosity increase reduces the alumina theoretical value (n_Al_~1.77) due to the increase in air into the nanopores, while pore size increase seems to eliminate its wavelength oscillatory dependence [39], which is a characteristic of photonic crystals [65]. Although differences depending on the IL can be observed, curves obtained for the NPAS/BMIMPF_6_ and the NPAS/OMIMPF_6_ samples are rather similar, in agreement with the weaker effect of the cation with respect to the anion exhibited by the ILs [38].

**Figure 5:**
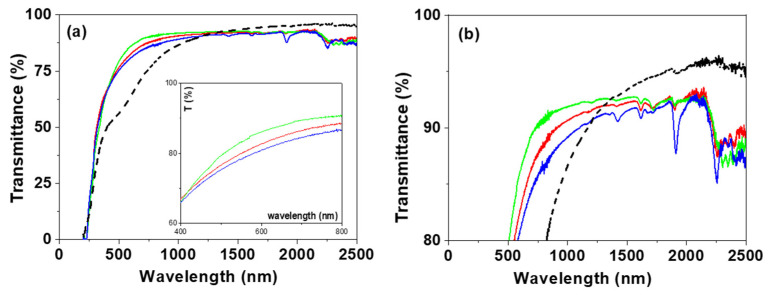
Light transmission versus wavelength for: (**a**) BMIMPF_6_/NPAS (red solid line), OMIMPF_6_/NPAS (green solid line) and EMIMBF_4_/NPAS (blue solid line) samples as well as the NPAS support (black dashed line); insert corresponds to visible region. (**b**) Enlargement of the transmission–wavelength curves.

Light transmittance and reflection measurements also give valuable optical information on the studied samples. Figure 5 shows the wavelength dependence of transmission percentage for the IL/NPAS samples, and, for comparison, the curve obtained for the NPAS support is also shown. As can be observed, significant differences in the transmission curves obtained for the IL/NPAS samples and the NPAS support were obtained for wavelengths ranging between 250 nm and 1200 nm, which seems to be associated with sample porosity [39]; however, differences in the visible region interval depending on the IL can also be observed in the insert in Figure 5a. Transmission % determined at four given wavelength values (600, 1000, 1500, and 2000 nm) for the IL/NPAS samples are indicated in Table 5, which clearly shows the similarity of the values in the NIR region. On the other hand, similitude in the band gap values for the three NPAS/IL samples (~228 nm) can also be observed from Figure 5a. The enlargement of the transmittance (%) axis shown in Figure 5b allows us to distinguish the different peaks shown in the nir region for the three IL/NPAS samples, which correspond to wavelengths around 1610 nm, 1905 nm, and 2262 nm (2256 in the case of EMIMBF_4_/NPAS), but another peak at 1420 nm can be observed for the EMIMBF_4_/NPAS one, being almost a general statement that the greatest depth in the peaks is exhibited by this latter sample (except in the case of that at 1715 nm). It should be indicated that absorption bands at around 1640 nm, 1720 nm, and 2150 nm for dry BMIMPF_6_ IL have already been reported, but several smaller bands in the region between 900 nm to 1500 nm for hydrated BMIMPF_6_ were also detected. Moreover, differences depending on the anions of the ILs were also observed, providing BF_4_^−^’s strongest interactions and PF_6_^−^ [66,67].

**Figure 6:**
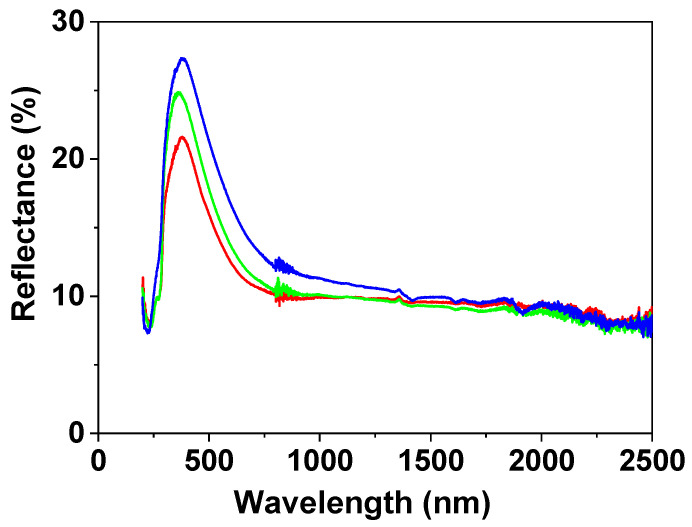
Light reflection versus wavelength for: BMIMPF_6_/NPAS (red solid line), BMIMPF_6_/NPAS (green solid line), and EMIMBF_4_/NPAS (blue solid line) samples.

Light reflection percentage, R (%), for the NPAS/IL samples was also measured and its variation with wavelength is shown in Figure 6, where differences in the visible region depending on the IL can be observed (although the three samples show a maximum at wavelength around 380 nm associated with the support) according to the following sequence: BMIMPF_6_/NPAS < OMIMPF_6_/NPAS < EMIMBF_4_/NPAS, but a similar reflection percentage in the nir region (~8% for wavelength higher than 1200 nm) was obtained for the three samples.

**Figure 7:**
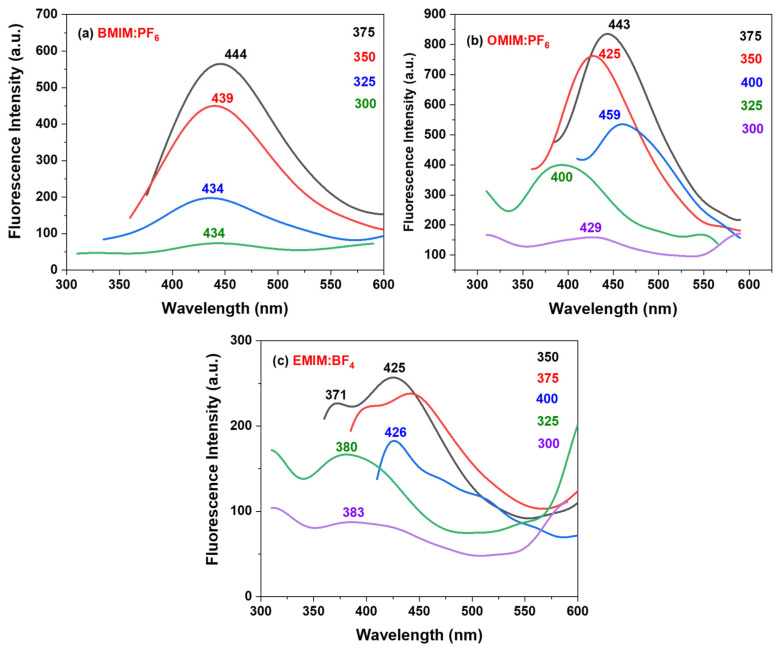
Fluorescence spectra of the selected ILs deposited on the surface of NPAS excited between 300 and 400 nm, with wavelength of maximum values indicated over each spectrum.

Fluorescence spectra of the ILs covering the surface of NPAS are depicted in Figure 7, which shows an emission with excitation wavelength dependence (between 300 and 400 nm) mainly when EMIMBF_4_/NPAS and OMIMPF_6_/NPAS samples are considered. Since the fluorescence character of nanoporous alumina structures has already been reported [68,69], irradiation of the used NPAS support with excitation wavelength ranging between 300 nm and 400 nm was also performed, but only dispersion was observed (see Figure S5 in Supplementary Information); this result could be associated with the asymmetry of the NPAS as well as the high pore size, porosity, and thickness of the open surface since photoluminescence of NPASs seems to increase when their hexagonal order is improved [70].

The fluorescence spectra for the BMIMPF_6_/NPAS sample (Figure 7a) show a unique band centered at around 445 nm when irradiated between 300 and 375 nm (particular values of the maximum for each curve are given into the figure), indicating that only one excited state is formed when irradiated under that range of wavelengths, which makes this sample of interest for future applications due to their unique optical behavior; this fact seems to be associated with the presence of the NPAS support since an almost linear increase was reported for the BMIMPF_6_ IL [71]. However, the OMIMPF_6_/NPAS sample shows most intense emission, with emission bands dependent on the excitation wavelength (for the same irradiation interval), showing emission between 395 and 460 nm (Figure 7b), which could be related to the different disposition of the OMIMPF_6_ molecules on the alumina surface. Similarly, the EMIMBF_4_/NPAS sample also exhibits excitation wavelength dependence (Figure 7c), showing a maximum of emission centered at 426 nm when excited at 350 nm with a shoulder around 371 nm, and similar behavior (with slightly less intensity) was found when the sample was excited at 375 nm (centered at 444 nm and with a shoulder at 399 nm). The differences between the main peak and the shoulder for both excitations are 55 nm and 45 nm respectively, which can indicate that two different excitation states could be found in the EMIMBF_4_/NPAS system; this fact is also an EMIMBF_4_/NPAS sample characteristic, since a linear increase with the excitation wavelength was also reported for the EMIMBF_4_ IL [17], and it could be related to the small size of the ethyl tail of this IL, provoking a different arrangement on the surface of the alumina nanosupport, in agreement with XPS experimental results and quantum chemistry modeling. In terms of emission intensity, the system OMIMPF_6_/NPAS presents the most interesting behavior, with 47.1% and 193% intensity increments when compared to BMIMPF_6_/NPAS and EMIMBF_4_/NPAS, respectively.

### 3.4. Solar Cell Application

**Figure 8:**
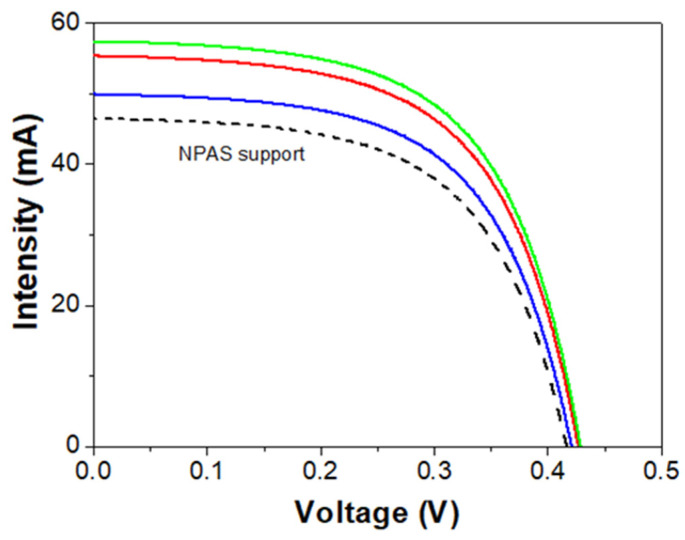
Intensity-voltage curves for the BMIMPF_6_/NPAS (solid red line), the OMIMPF_6_/NPAS (solid green line), and the EMIMBF_4_/NPAS (solid blue line) systems and the NPAS support (dashed black line).

This section presents the I–V curve results obtained by over-positioning the IL/NPAS systems on a monocrystalline silicon solar cell using the NPAS support as reference. Figure 8 shows the I–V curves for the three IL/NPAS systems and the NPAS support, while the average values of the main parameters, short circuit current (I_sc_), and power at the maximum power point (P_mp_), obtained from the I–V curves, are shown in Table 6. Values determined for other characteristic parameters (open circuit voltage, V_oc_, fill factor, FF, power absolute difference, and the relative difference in power) are given as Supplementary Information (Table S2).

As it can be observed in Figure 8, the three IL/NPAS systems show an increase in the I_sc_ value when compared to the NPAS substrate, and a quantitative analysis can be performed by considering the results shown in Table 6: the short circuit current values obtained for the BMIMPF_6_/NPAS and the OMIMPF_6_/NPAS systems are rather similar and more than 10% higher than that determined for the EMIMBF_4_/NPAS one. In fact, the three IL/NPAS systems improve the short circuit current value with respect to the NPAS support (between 9% and 19%), having a direct impact over the output power of the three IL/NPAS systems (increase between 1.44% and 3.34%), and with the same sequence: OMIMPF_6_/NPAS ≥ BMIMPF_6_/NPAS > EMIMBF_4_/NPAS.

The behavior exhibited by the IL/NPAS systems is directly related to the emissions presented by them, since any increase in the radiation generated in the range of response of the monocrystalline solar cell has a positive effect on the photo response [72]. However, its impact, i.e., the increase in the parameters of the I–V curve, depends on: (i) the intensity, since more photons can be transformed into photocurrent, and (ii) the position of this emission, because they must have enough energy to give rise to the photovoltaic effect (for monocrystalline silicon, positioned between 350 nm and 1100 nm). This explains why the three IL/NPAS systems improve the photo response of the cell since, independently of the laser excitation used, all the samples emit in the range of the electromagnetic spectrum between 350 and 550 nm (although not equally). It should be pointed out that the EMIMBF_4_/NPAS system exhibits the best photo response, since it presents an important emission for all excitations, especially from 350 nm onwards; in other words, a significant part of the ultraviolet spectrum is used to generate extra photons and, in the case of the spectrum obtained with the 400 nm laser, it shifts slightly to longer wavelengths, close to the optimum of photovoltaic conversion (≥500 nm). The second-best responding system is the BMIMPF_6_/NPAS; since its emission is significant only for excitation lasers starting at 350 nm, then it could provide extra solar radiation only from wavelengths > 350 nm but without achieving the level reached by EMIMBF_4_/NPAS and positioned slightly towards lower wavelengths. However, the OMIMPF_6_/NPAS system exhibits the poorest behavior for commercial silicon solar cell technology applications since it has the lowest intensity emission for all the excitation lasers, shifting towards shorter wavelengths, that is, the region of the spectrum where the solar cell responds more poorly. In this context, the selection of nanoporous alumina supports with tunneled geometrical and surface characteristics (by proper election of fabrication parameters), able to provide them photoluminescence character in the adequate region of the electromagnetic spectrum, could be of interest and they will be considered in the near future.

## 4. Conclusions

Modification of the optical properties of a nanoporous alumina support (NPAS) by deposition of a layer of different imidazolium-based ionic liquids (BMIMPF_6_, OMIMPF_6_ or EMIMPF_6_) was established. XPS analysis of the IL/NPAS samples shows the almost total coverage of the support as well as anion–alumina interactions, in agreement with quantum chemical analysis which indicates that the EMIMBF_4_/NPAS system exhibits the most stable bond between the ILs and the NPAS.

Optical characterization of the IL/NPAS samples indicates high transparency for all of them (>80% in the visible region and >90% in the near-infrared one), while the value of the refractive index determined from SE measurements hardly differs from the corresponding IL, with these results corresponding to a wider range of wavelengths (250–1700 nm). The three IL/NPAS samples exhibit photoluminescence with the following intensity sequence: OMIMPF_6_/NPAS > BMIMPF_6_/NPAS > EMIMBF_4_, and maximums for the emission bands ranging between 400 nm and 460 nm (central part of the electromagnetic spectrum), opening its application to solar cells. In fact, the electrical characterization of the three IL/NPAS systems indicates an improvement in photovoltaic conversion, between 1.44% and 3.34%, with the sequence OMIMPF_6_/NPAS ≥ BMIMPF_6_/NPAS > EMIMBF_4_/NPAS. The use of NPASs with fluorescence behavior could improve these figures.

## Figures and Tables

**Table 1:** Physicochemical parameters of the selected ILs.

ILs	Molecular Weight (g/mol)	Molar Volume (cm^3^/mol)	Density (kg/m^3^)	Viscosity (mPa·s)	Refractive Index *
BMIMPF_6_	284.2	182	1.38 × 10^3^	229	1.409
OMIMPF_6_	340.3	266	1.23 × 10^3^	522/1000	1.417
EMIMBF_4_	197.9	159	1.29 × 10^3^	76/108	1.405

* [38].

**Table 2:** Atomic concentration percentages of the elements found on the surfaces of the IL/NPAS samples. Experimental and theoretical (in italics) atomic concentration ratio of different elements.

**Sample**	**C (%)**	**N (%)**	**F (%)**	**P (%)**	**B (%)**	**Al (%)**	**O (%)**
BMIMPF_6_/NPAS	4 48.8	7.4	24.8	5.5	----	5.1	8.3
OMIMPF_6_/NPAS	5 55.4	7.2	21.4	5.0	----	3.9	7.0
EMIMBF_4_NPAS *	6 63.5	6.6	14.3	----	5.6	3.1	6.1
**Sample**	**(C/N) ^E^ (C/N) ^T^**	**(F/N) ^E^ (F/N) ^T^**	**(C/F) ^E^ (C/F) ^T^**	**(F/P) ^E^ (F/P) ^T^**	**(F/B) ^E^ (F/B) ^T^**	**(O/Al) ^E^(O/Al) ^T^**	
NPAS/BMIMPF_6_	4 6.6 4.0	3.4 3.0	2.0 1.3	4.5 6.0	- -	1.6 1.5	
NPAS/OMIMPF_6_	5 7.7 6.0	3.0 3.0	2.6 2.0	4.3 6.0	- -	1.8 1.5	
NPAS/EMIMBF_4_	6 9.6 3.0	2.2 2.0	4.4 1.5	- -	2.6 4.0	2.0 1.5	

(E: Experimental and T: Theoretical). * [38]

**Table 3:** Formation electronic energies in kcal/mol of the imidazole moiety/NPAS systems.

System	Δ_a_E (Kcal/mol)
BMIMPF_6_/NPAS	−59.5
OMIMPF_6_/NPAS	−89.8
EMIMBF_4_/NPAS	−97.0

**Table 4:** Average values of refractive index (n) for the studied samples for the visible (v) and near-infrared (nir) regions.

Sample	<n_v_>	<n_nir_>
BMIMPF_6_/NPAS	1.467 ± 0.006	1.437 ± 0.013
OMIMPF_6_/NPAS	1.480 ± 0.010	1.445 ± 0.003
EMIMBF_4_/NPAS	1.461 ± 0.029	1.408 ± 0.002

**Table 5:** Transmittance percentage at specific wavelength values for the IL/NPAS samples.

Sample	T % (600 nm)	T % (1000 nm)	T % (1500 nm)	T % (2000 nm)
BMIMPF_6_/NPAS	82.3	91.0	92.1	92.3
OMIMPF_6_/NPAS	86.2	91.6	91.5	92.4
EMIMBF_4_/NPAS	80.5	89.5	91.5	92.0

**Table 6:** Average values of short circuit current (I_sc_) and power at the maximum power point (P_mp_) determined for the three IL/NPAS systems.

Sample	I_sc_ (mA)	P_mp_ (mW)
NPAS	46.44	11.38
BMIMPF_6_/NPAS	55.15	14.08
OMIMPF_6_/NPAS	57.27	14.74
EMIMBF_4_/NPAS	50.53	12.67

## Data Availability

Not applicable.

## Supplementary Materials

The following supporting information can be downloaded at: https://www.mdpi.com/article/10.3390/nano12234131/s1, Figure S1. Scheme of incident, reflected, and transmitted light for a two-layer sample and SE experimental Ψ and Δ angle relationships. Figure S2. XPS survey spectra for the studied samples. Figure S3. Core level signals of B *1s* and P *2s.* Figure S4. Light wavelength dependence of tan (Ψ) and cos (∆). Figure S5. Fluorescence spectra of the NPAS support. Table S1. Average atomic concentration percentages. Table S2. Open circuit voltage, fill factor, power absolute difference, and relative difference in power.
