# Supplementary material for: Nanoporous Alumina Support Covered by Imidazole Moiety–Based Ionic Liquids: Optical Characterization and Application
Source: Nanomaterials (Basel). 2022 Nov 23;12(23):4131. doi: 10.3390/nano12234131 (PMC9736403; doi:10.3390/nano12234131)
Supplement: Supplementary file 1 [file nanomaterials-12-04131-s001.zip › nanomaterials-2027904-supplementary.pdf]

Supplementary Information

Nanoporous Alumina Support Covered by Imidazole Moiety–Based Ionic Liquids: Optical Characterization and Application

Manuel Algarra [1,*], Mª Cruz López Escalante [2], Mª Valle Martínez de Yuso [3], Juan Soto [4], Ana L. Cuevas [5] and Juana Benavente [5,*]

[1] INAMAT²-Institute for Advanced Materials and Mathematics, Departamento de Ciencias, Universidad Pública de Navarra, Campus de Arrosadía, 31006 Pamplona, Spain.
[2] Departamento de Ingeniería Química, Facultad de Ciencias, Universidad de Málaga, 29071 Málaga, Spain; mclopez@uma.es
[3] X-ray Photoelectron Spectroscopy Lab., Central Service to Support Research Building (SCAI), University of Málaga, 29071 Málaga, Spain; mvyuso@uma.es
[4] Departamento de Química-Física, Facultad de Ciencias, Universidad de Málaga, 29071 Málaga, Spain; soto@uma.es
[5] Unidad de Nanotecnología, Centro de Supercomputación y Bioinnovación, Servicios Centrales de Investigación, Universidad de Málaga, 29071 Málaga, Spain; analaura.cuevas@uma.es
[6] Departamento de Física Aplicada I, Facultad de Ciencias, Universidad de Málaga, 29071 Málaga, Spain; j_benavente@uma.es
* Correspondence: manuel.algarra@unavarra.es (M.A.); j_benavente@uma.es (J.B.).

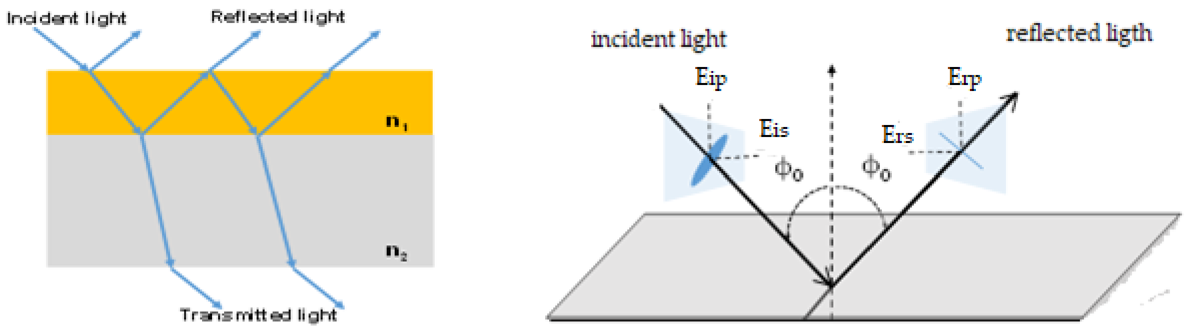

(a) (b)

Figure S1. (**a**) Scheme of incident, reflected transmitted light for a two layers sample. (**b**) SE experimental Ψ and Δ angles, which are related with the amount of reflected light polarized in the perpendicular plane with respect to the incidence light plane (r_P) and the amount of reflected light polarized in a plane parallel to the incidence light plane (r_s), being $\tan(\Psi)\times e^{i\Delta} = r_p/r_s$, where tan(Ψ) is the amplitude ratio upon reflection and Δ is the phase shift difference [52].

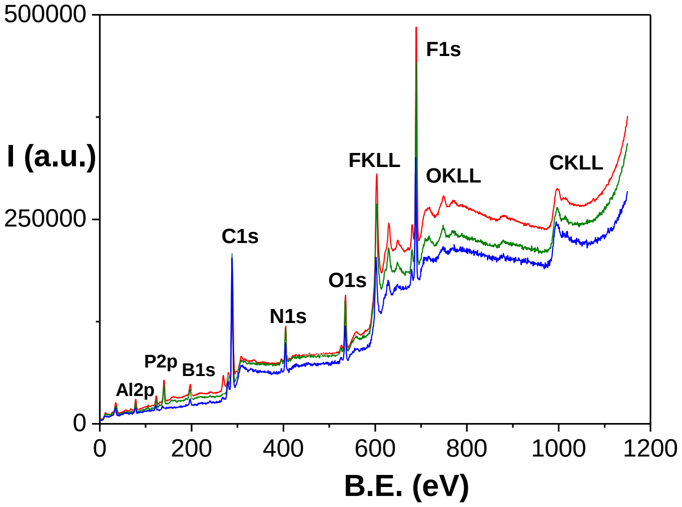

Figure 2. Survey XPS spectra for: BMIMPF₆/NPAS (red line), OMIMPF₆/NPAS (green line), and EMIMBF₄/NPAS (blue line) samples.

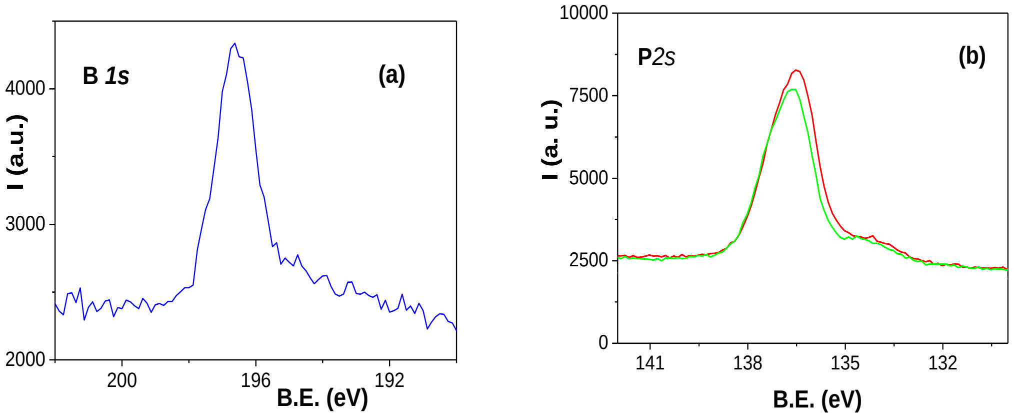

Figure S3. Core level signals of: (**a**) B *1s* for EMIMBF₄/NPAS sample; (**b**) P *2s* for BMIMPF₆/NPAS (red line) and OMIMPF₆/NPAS (green line) samples.

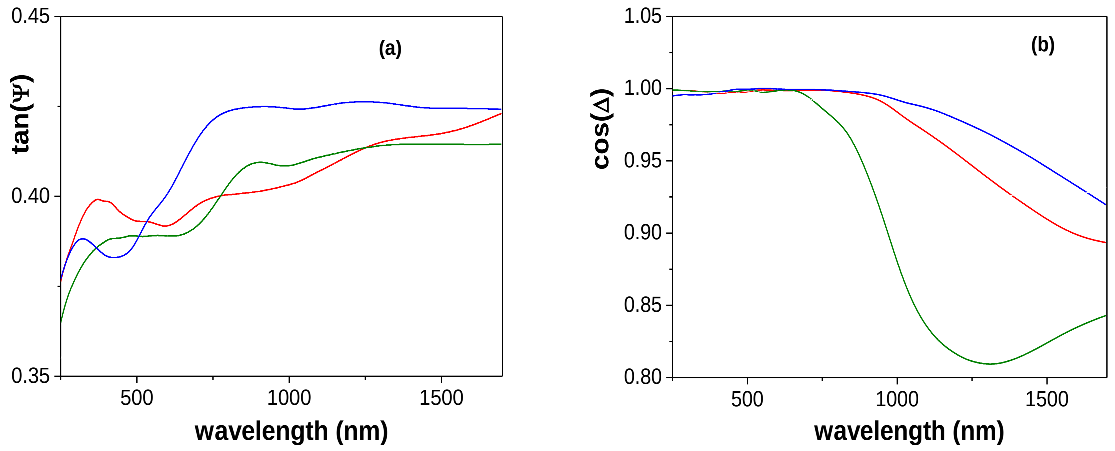

Figure S4. Light wavelength dependence of: (**a**) tan(Ψ) and (**b**) cos(Δ), for NPAS/$BMIMPF_6$ (red line), NPAS/$OMIMPF_6$ (green line) and NPAS/$EMIMBF_4$ (blue line) samples.

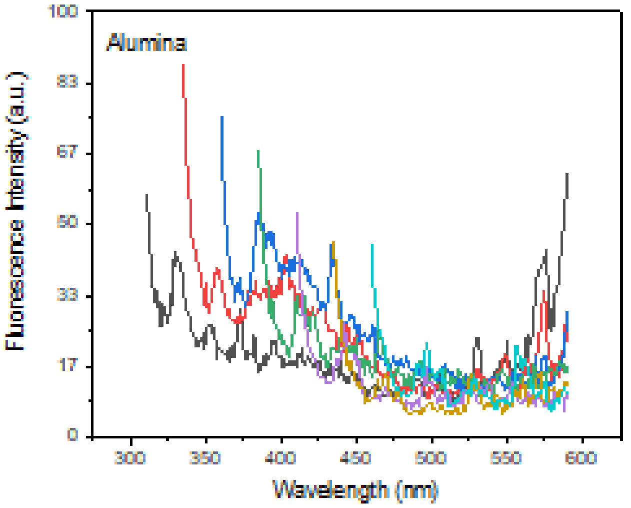

Figure S5. Fluorescence spectra of the NPAS support at different excitation wavelengths.

Table S1. Average atomic concentration percentages of the main chemical elements found on the surface of the NPAS [42].

Sample	C (%)	O (%)	Al (%)	N (%)	S (%)
NPAS	16.6	50.8	27.8	0.5	3.1

Table S2. Average values of open circuit voltage (V_{oc}), fill factor (FF), power absolute difference of and relative difference in power and with respect the reference.

Sample	V_{oc} (mV)	FF (%)	Difference P_{mp} (mW)	Relative Difference P_{mp} (%)
NPAS	416	58.88		
$BMIMPF_6$/NPAS	428	59.75	2.71	23.79
$OMIMPF_6$/NPAS	430	59.98	3.36	29.53
$EMIMBF_4$/NPAS	422	59.42	1.29	11.36
